# Long-Term Viral Brain-Derived Neurotrophic Factor Delivery Promotes Spasticity in Rats with a Cervical Spinal Cord Hemisection

**DOI:** 10.3389/fneur.2013.00187

**Published:** 2013-11-19

**Authors:** Karim Fouad, David J. Bennett, Romana Vavrek, Armin Blesch

**Affiliations:** ^1^Rehabilitation Medicine, University of Alberta, Edmonton, AB, Canada; ^2^University of Alberta, Edmonton, AB, Canada; ^3^Spinal Cord Injury Center, University Hospital Heidelberg, Heidelberg, Germany; ^4^University of California, San Diego, La Jolla, CA, USA

**Keywords:** spinal cord injury, BDNF, combined treatment, TrkB-Fc, EMG

## Abstract

We have recently reported that rats with complete thoracic spinal cord injury (SCI) that received a combinatorial treatment, including viral brain-derived neurotrophic factor (BDNF) delivery in the spinal cord, not only showed enhanced axonal regeneration, but also deterioration of hind-limb motor function. By demonstrating that BDNF over-expression can trigger spasticity-like symptoms in a rat model of sacral SCI, we proposed a causal relationship between the observed spasticity-like symptoms (i.e., resistance to passive range of motion) and the over-expression of BDNF. The current study was originally designed to evaluate a comparable combined treatment for cervical SCI in the rat to improve motor recovery. Once again we found similar signs of spasticity involving clenching of the paws and wrist flexion. This finding changed the focus of the study and, we then explored whether this spasticity-like symptom is directly related to the over-expression of BDNF by administering a BDNF antagonist. Using electromyographic measurements we showed that this treatment gradually diminished the resistance to overcome forelimb flexion in an acute experiment. Thus, we conclude that neuro-excitatory effects of chronic BDNF delivery together with diminished descending control after SCI can result in adverse effects.

## Introduction

Axonal regeneration in the adult mammalian central nervous system is inhibited by numerous factors (1) impeding the development of effective treatments for brain or spinal cord injuries (SCI). Consequently, it is likely that repair of the injured spinal cord by axonal regeneration and plasticity will require a combined treatment approach. Several combinatorial strategies have been pursued with a wide range of outcomes [e.g., Ref. (2–5)]. These experiments are not only technically demanding, but also challenging in the interpretation of the results, considering the unpredictable effects of promoting neurite outgrowth and possible interactions between treatment components (6, 7).

One prominent molecule that has been the subject of many studies in spinal cord repair is brain-derived neurotrophic factor (BDNF). BDNF not only has neuro-protective, regeneration, and plasticity promoting effects but also neuro-excitatory properties and binds with high affinity to the TrkB receptor and with lower affinity to p75 [reviewed in Ref. (8)]. As such, BDNF is a prominent candidate for combined treatment approaches [e.g., Ref. (6, 9, 10)]. However, due to the broad range of its effects, the consequences of BDNF delivery can be complex. For example, we observed that a combinatorial treatment involving BDNF (achieved by direct viral vector injection and bone marrow stromal cell grafts genetically modified to express BDNF) promoted spasticity in rats with a cervical hemisection. Because the combined treatment also promoted axonal regeneration and plasticity, the origin of the observed spasticity remains unclear (11). We could, however, provide an indirect explanation by demonstrating that the over-expression of BDNF could promote the development of hyperreflexia in tail muscles in a sacral model of SCI (11). Yet, it remains unclear whether this effect of BDNF was caused by its neuro-excitatory properties or through an augmentation of neuroplasticity and/or regenerative growth. However, this was not the original question of the current study. We initially set out to test a combined treatment that addressed multiple limitations for neurite growth following cervical SCI. The focus of the analysis shifted when severe spasticity-like symptoms in the injured forelimb became evident, which led us to focus on determining the cause of spasticity. To address this question we administered a BDNF antagonist into the spinal cord of rats that showed spasticity-like symptoms, and these were gradually reduced by this treatment.

## Materials and Methods

### Animals

Adult female Fischer 344 rats (Charles River, 180–220 g) were group housed and kept at 12/12 h light/dark cycle with *ad libitum* water. The study was approved by the Animal Care and Use Committee for Health Sciences of the University of Alberta, and complies with the guidelines of the Canadian Council for Animal Care.

### Lesion surgery and treatment

Rats were anesthetized (using Hypnorm 0.16 mg/kg; Vetapharma, Leeds, UK; and Midazolam 2.5 mg/kg; Sandoz, Canada; diluted in sterile water) and mounted into a stereotaxic frame (Kopf Instruments), and the spinal cord between C5 and C6 was exposed by performing a laminectomy of half of the C5 segment. The spinal cord was lesioned using a microsuction pipette and a spring scissor. A lateral hemisection lesion was performed ipsilateral to the preferred paw as determined by a forelimb reaching task. A small gap was created to ensure lesion completeness and provide space for the cell graft. The dura was sealed with a thin agarose film (Sigma) and fibrin glue (Baxter, USA), overlying muscles were sutured and the skin was stapled. Rats were placed on a heating pad until they were awake and received buprenorphine (0.03 mg/kg) as analgesic and saline over the next 2 days.

#### Preparation of cell grafts to provide a tissue bridge for regenerating axons

Fibroblasts were isolated from skin biopsies of Fischer 344 rats and cultivated in D’MEM/10% FBS with antibiotics. Cells were transduced with retroviral vectors to express BDNF as described by Lu et al. (11, 12) or NT-3 (13) and selected for G418 resistance. BDNF and NT-3 expression was measured *in vitro*, in 24 h cell culture supernatants by ELISA as described (14, 15). BDNF-transduced fibroblasts expressed 27 ng/10^6^ cells/24 h, NT-3 transduced cells expressed 134 ng/10^6^ cells/24 h. For grafting, BDNF and NT-3 expressing cells were mixed 1:1 and 2–3 μl were injected into the lesion site using a 5-μl Hamilton syringe at a concentration of 2.5 × 10^4^ cells/μl.

#### scAAV vector preparation and injections

Self-complementary adeno-associated viral vectors (scAAV) were generated as described by Lu et al. (11). In order to attract regenerative growth out of the graft into the caudal spinal cord, BDNF, and NT-3 (and GFP as control) transfected scAAV vectors (1.3 μl/site) were injected 1.25, 2.5, and 4 mm caudal to the lesion on the side ipsilateral to the lesion through pulled glass capillaries using a Picospritzer II similar to Lu et al. (11). The injection volume was divided over a depth of 1 and 1.5 mm.

One week post-lesion surgery, cell grafts and viral vector injection surgeries were performed in all lesioned animals in the following groups:

Group 1 is a CONTROL group used to demonstrate the ability of self-complementary adeno-associated virus (scAAV) vectors to successfully infect spinal neurons. This group did not receive a cell graft, only PBS, followed by injections of 4.3 × 10^11^ vg/ml scAAV vectors expressing Green Fluorescent Protein (scAAV2-GFP) at 1.25, 2.5, and 4 mm caudal to the lesion; *n* = 16.
Group 2, scAAV-BDNF group received an injection of PBS in the lesion site, followed by 4.3 × 10^11^ vg/ml scAAV-BDNF injections at 1.25, 2.5, and 4 mm caudal to the lesion to serve as trophic support for regenerating fibers; *n* = 5.
Group 3, the GRAFT/BDNF/NT-3 or FULL treatment group, received a fibroblast cell graft (expressing BDNF and NT-3) into the lesion site to provide a permissive cellular matrix that allows axonal growth, and a trophic stimulus to stimulate axonal growth. This group also received injections of a mixture of scAAV2 BDNF (f.c. 4.3 × 10^11^ vg/ml), scAAV2-NT-3 (f.c. 4.3 × 10^11^ vg/ml), and scAAV2-GFP (f.c. 4 × 10^10^ vg/ml) mixed with 20 U/ml ChABC (Seigaku, USA) caudal to the lesion (1.25, 2.5, and 4 mm). The neurotrophic mixture is intended to provide trophic stimulus beyond the lesion and ChABC degrades growth inhibitory proteoglycans in the scar tissue (3). In addition, this group also received a subcutaneous injection of the phosphodiesterase (PDE) inhibitor, Rolipram, (AG Scientific, San Diego, CA, USA; 3 ml of 3 mg/kg in 2% DMSO and saline) once per day for 14 days following the lesion. This drug elevates cAMP levels in the CNS and augments regeneration-related gene expression in injured neurons (16); *n* = 18.
Group 4, the GRAFT/scAAV-NT-3 group received a fibroblast cell graft expressing BDNF and NT-3 into the lesion site, injections of scAAV expressing NT-3 and GFP only, mixed with ChABC caudal to the lesion and subcutaneous injections of rolipram; *n* = 7.
Group 5, the GRAFT/ChABC group received a fibroblast cell graft expressing BDNF and NT-3 into the lesion site and ChABC injections 1.25, 2.5, and 4 mm caudal to the lesion, followed by daily rolipram administration; *n* = 5.

### Behavioral testing

#### Single-pellet grasping task

This test was performed as described previously (17, 18). In order to motivate rats to grasp for sugar pellets (45 mg, banana flavor; TestDiet, Richmond, CA, USA) in the task, restricted amounts of food were allowed directly after the daily training session (10 g/rat/day). A transparent Plexiglas chamber (30 cm × 36 cm × 30 cm) with a narrow opening in the front wall and an attached shelf outside (2 cm distance from the inside of the front wall, 3 cm above the floor) was used. Pellets were placed at a distance such that the rats had to grasp them with their preferred forelimb and could not reach them with their tongue. The preferred paw was determined for each rat during the first few training sessions. Successful grasps were scored over a 10-min period only when a rat was able to grasp the presented pellet, bring it to its mouth and eat it. Pre-operative baseline values were obtained after a 4-week training period (five times per week, 10 min per rat). Post-operative rehabilitative training of the injured forelimb started 1 week after graft implantation surgery (2 weeks post-lesion).

#### Forelimb placement

**Figure 1:**
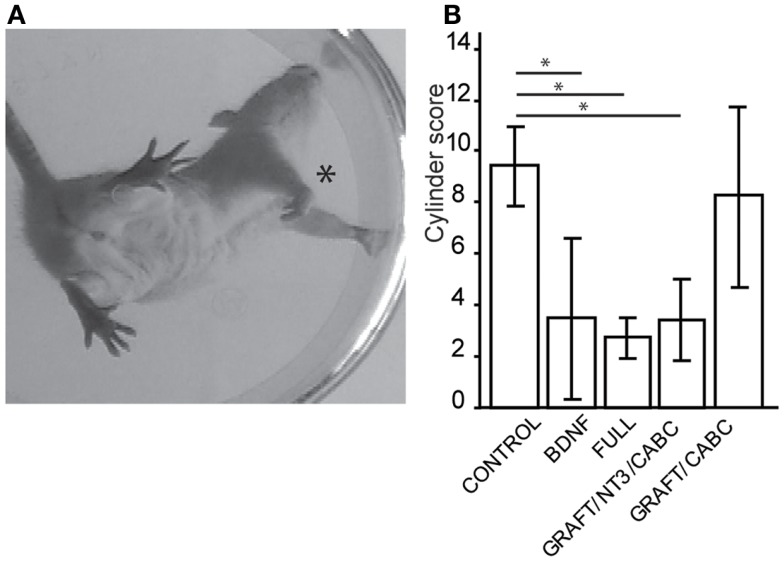
**Treatments involving BDNF promoted exaggerated wrist flexion and affected forelimb use**. Signs of wrist flexion became apparent as early as 2 weeks after treatment **(A)**. By 9 weeks post-injury the cylinder exploration task indicated that animals in the FULL treatment group and the scAAV-BDNF only group had significantly reduced use of the injured forelimb (*) when compared to control animals **(B)**. Error bars show average ± SE; *p < 0.05, **p < 0.005, ***p < 0.0005.

To further assess forelimb function, all rats were scored over a 4-min period in a Plexiglas open field according to Martinez et al. (19), with scores ranging from 0 (no function) to 20 (no deficit). Behavioral components were categorized from global fore- and hind-limb movements to antero-posterior coordination, which included weight support, fine distal positioning, and stepping abilities. Nine weeks post-lesion, asymmetries in spontaneous forelimb use were evaluated by placing individual rats into a clear Plexiglas cylinder (24 cm high/19 cm inner diameter). A mirror was placed underneath the cylinder at an angle to facilitate the videotaping of the rat’s vertical exploratory activity in the cylinder (Figure 1). Ten rearing movements were recorded in each test session. Each forepaw placement on the wall of the cylinder was scored during a rearing movement. If the affected forepaw was plantar on contact with the wall of the cylinder, the number of contacts was multiplied by two, if only the dorsal surface of the paw was placed on the wall, the number of contacts was multiplied by one and if the paw did not contact the wall a score of 0 was assigned, thus allowing for a maximum score of 20.

#### Grip strength

**Figure 2:**
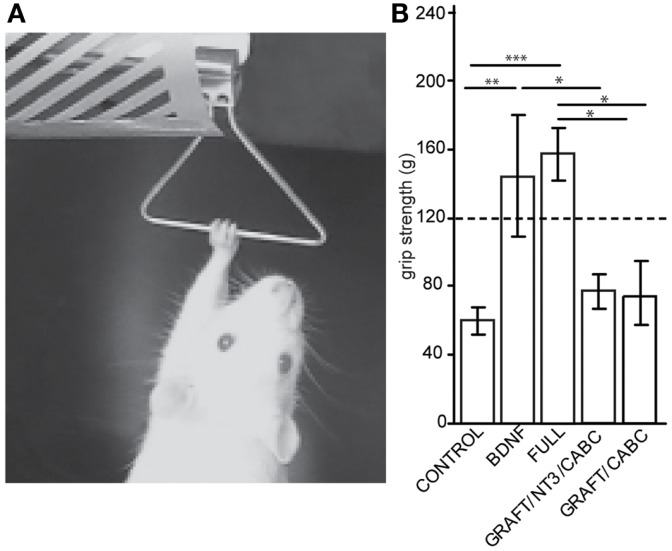
**Brain-derived neurotrophic factor expression increases wrist flexion: using an apparatus designed to measure grip strength (A) we compared measurements between control animals and the rats receiving scAAV-BDNF only or the FULL treatment (B)**. When compared with the control group, a significant increase in “grip” strength was observed in both the scAAV-BDNF and the FULL treatment groups at 9 weeks post treatment. The dashed line indicates a normal grip strength value. Error bars show average ± SE; **p < 0.01, ***p < 0.001.

The grip strength of the forelimbs (or in other words, the force needed to overcome wrist and digit flexion when actively or passively holding on to a bar) was assessed 9 weeks post-lesion by measuring the maximum force (in gram) exerted by each forelimb, with a Grip Strength Meter apparatus (Columbus Instruments, OH, USA, Figure 2). For the final values, four readings for both paws were obtained over three different trials and averaged for post-operative analysis.

#### Sensory test

Forelimb withdrawal latency to a heat stimulus was measured prior to injury (baseline) and 9 weeks post-lesion using methods previously described (20). Briefly, the rats were placed on a glass plate over a light box, and a radiant heat stimulus (Ugo Basile) was applied by aiming a beam of light onto the plantar surface of the paw of each forelimb through the glass plate. The light beam was turned off automatically when the rat lifted the limb, allowing the measurement of time between the start of the light beam and the paw withdrawal. Five minutes were allowed between three trials, baseline values were averaged for each limb and compared to post-injury values.

### Electrophysiology

Electromyographic (EMG) recordings of flexor muscles of the injured paw were carried out prior to perfusion. Animals were anesthetized with 5% isoflurane gas anesthesia for induction and 1.5% for maintenance of anesthesia and placed into a stereotaxic frame. The spinal cord surrounding the hemisection lesion was exposed. To record EMG responses during manual wrist extension, two electrodes with exposed tips (Teflon coated wire; A-M Systems, Carlsborg, WA, USA) were inserted into wrist flexors of the forelimb ipsilateral to the lesion. The EMG signal was amplified (Grass, Astro-Med Inc., West Warwick, RI, USA), digitized (5 kHz, Digidata 1322A; Axon instruments, Foster City, CA, USA), and filtered (30–300 Hz) as described earlier (17). The muscle responses were recorded under anesthesia before and 10, 20, and 30 min following injection of the TrkB antagonist, TrkB-Fc (1 μl of 0.2 mg/ml; Sigma) or saline into the spinal cord lesion site.

The following animals were selected for EMG analysis: (i) three rats without signs of spasticity (one from the CONTROL group and two from the FULL treatment group) received a TrkB-Fc injection. (ii) Four rats with spasticity (one from the scAAV-BDNF, two from the FULL treatment group and one from GRAFT/NT-3) received a saline injection only. (iii) Ten rats with spasticity (two from scAAV-BDNF and eight from FULL treatment group) received TrkB-Fc injection.

Electromyographic responses were recorded, and the burst duration was measured using Axoscope software (Axoscope 9.0.1.16; Molecular Devices, Sunnyvale, CA, USA). The traces were rectified and the average burst amplitude was quantified and then multiplied by the burst duration using Microsoft Excel.

The animals were euthanized immediately following the EMG recordings with pentobarbital (Euthanyl, Bimeda-MTC; 70/100 mg body weight) and transcardially perfused with phosphate-buffered saline, followed by 4% paraformaldehyde (PFA) in 0.1 M phosphate buffer, pH 7.4. The spinal cord was removed, post-fixed in 4% PFA overnight at 4°C and cryo-protected in 30% sucrose over 2 days. The cervical enlargement encompassing the lesion site (C2–6) was embedded in Tissue Tek (Sakura Finetek USA Inc., Torrance, CA, USA) and frozen in 2-methyl-butane at −60°C.

### Histology

Spinal cords were cryosectioned in the horizontal plane at 25 μm and mounted in eight series onto Fisherbrand slides (Fisher Scientific, Ottawa, ON, Canada). To evaluate the lesion site, sections were counterstained with 0.1% Cresyl Violet and dehydrated in increasing alcohol concentrations, cleared with xylene, and coverslipped with Permount (Fisher Scientific). Lesion sizes were reconstructed from C2 to C6 spinal cord tissue by analyzing every fourth section through the dorso-ventral plane of the spinal cord by using central canal and gray/white matter interphases as landmarks. From these reconstructions, the percent cross-sectional surface area of the damaged tissue was measured using Image J (NIH) software. Animals with lesions deviating from a complete hemisection by >10% were removed from this study.

Immuno-histochemistry of serotonergic (5-HT) fibers was carried out on every second horizontal section encompassing the lesion site using rabbit anti 5-HT antibody (S5545, Sigma-Aldrich, Oakville, ON, Canada). In brief, following a 10% normal goat serum (NGS, Vector Labs, Burlingame, CA, USA) blocking step, the slides were incubated in 1:1000 dilution of primary antibody containing 1% NGS overnight at 4°C. The slides were then washed in TBS before an overnight incubation at 4°C in the secondary antibody (Vector Labs, Burlingame, CA, USA). Application of ABC and DAB solutions according to manufacturer’s recommendations followed (Vector Laboratories, Burlingame, CA, USA). After three washing steps of 10 min in TBS, the slides were serially dehydrated with alcohol, cleared with xylene, and coverslipped in Permount (Sakura Finetek USA, Torrance, CA, USA).

The density of 5-HT fibers rostral and caudal of the lesion was quantified from images taken at 400× using a Leica light microscope. Images were saved as 8-bit gray-scale TIFF files and imported into Image J software version 1.43q (NIH), where threshold values were adjusted. Selected threshold values were kept constant for all images to standardize the amount of background included in quantification. Integrated density measurement, representing the sum of the values of the pixels in an image, was averaged from five different images.

### Statistics

To determine statistical differences between treated and untreated animals, Student’s *t*-test and a one-way ANOVA with Tukey’s honestly significant difference *post hoc* test (Prism, V 4.0; GraphPad, San Diego, CA, USA) were used. All results and figures are presented as means ± SE of the mean. Statistical significance is stated for *p* values <0.05.

## Results

### Functional recovery

The post-lesion recovery process was overshadowed by the appearance of signs of spasticity in the forelimb ipsilateral to the lesion. The first signs involved clenching of the paw and especially pronounced wrist flexion that appeared 2 weeks post-injury. *Post hoc* analysis indicated that these signs of spasticity occurred in animals in all treatment groups. However, by 6 weeks, only 12% of rats (2 out of 16) in the control treatment group showed abnormal wrist flexion and clenching of the paw. This stood in contrast to 60% (3 out of 5) of rats that were injected with scAAV-BDNF and 72% (13 out of 18) of rats belonging to the FULL treatment group (involving cell grafts expressing BDNF and NT-3 in the lesion site and scAAV-BDNF expression caudal to lesion) and three out of seven rats (43%) in the GRAFT/NT-3 group which received the cell grafts expressing BDNF and NT-3 in the lesion site and scAAV-NT-3 only expression caudal to lesion. In the, GRAFT/ChABC group, consisting of cell grafts expressing BDNF and NT-3 and ChABC injection, only one out of five rats (20%) displayed signs of spasticity. These effects made further functional testing difficult. Rats with these signs of spasticity were unable to plantar step or to grasp for food pellets. Consequently, because most animals were unable to retrieve pellets, the single-pellet task had to be abandoned entirely. Although rats in all groups attempted to reach for pellets, a significant decline in the attempt rate was apparent in the treated groups when compared to their baseline attempt rate measurements (FULL treatment group: 15.4%, scAAV-BDNF group: 35%, GRAFT/NT-3 group: 56.7%; GRAFT/ChABC group: 25.7%, and control group: 86.7%).

A test capable of quantitatively assessing the deficits in hand/wrist function was the cylinder test (Figure 1A). When exploring the wall with their paws, rats showing signs of spasticity did not use the affected limb to explore, significant deficits were observed in the group receiving scAAV-BDNF only (3.5 ± 1.3), in the group receiving graft expressing BDNF/NT-3 and scAAV-NT-3 expressing vectors (3.4 ± 1.6), as well as in the FULL treatment group (2.7 ± 0.9) when compared to control group (9.4 ± 1.5) or the graft expressing BDNF/NT-3 and ChABC treated group (8.25 ± 3.4; Figure 1B).

When quantifying the forelimb deficits using a forelimb score [Ref. (19); a score of 0 indicates no forelimb function and 20 indicates no deficit] rats in the group receiving scAAV-BDNF only performed somewhat worse than control treated rats (9.2 ± 0.6 vs. 11.2 ± 0.8); however this difference was not found to be statistically significant. When controls were compared to the rats receiving the FULL treatment with markedly more cells expressing BDNF (due to the graft) a significant decrease in the performance was found (7.3 ± 0.5; *p* = 0.0008; data not shown).

A test that dramatically illustrates the inability to open the paw and the rigidity of the wrist flexors in these rats is the grip strength test (Figure 2A). By sliding the paw over the bar of the force sensor, the paw basically “hooked” onto the bar and the animal could be pulled away from the sensor in order to measure the force needed to overcome flexion and to release the bar. When comparing these forces between the groups for the unaffected arm (contralateral to the lesion), no differences were found. This is of interest considering that a potential neuro-excitatory effect of BDNF could spread over the entire spinal cord. Because the values were comparable between all groups, an average value from all animals (118.2 ± 4 g) is indicated in Figure 2B as “normal” grip strength.

When testing the affected limb we found that control animals with hemisection lesion (and no treatment) usually exert a moderate to weak resistance when being pulled away from the bar. The force necessary before releasing/sliding the affected paw over the bar was significantly lower than that for the unaffected paw(s) (60.1 ± 31.5 vs. 118.2 ± 4 g; *p* = 0.022). In contrast, rats with signs of spasticity were hardly able to release the bar resulting in higher values than those in the unaffected paw and significantly higher than the control treated rats on the affected side (scAAV-BDNF vs. Control *p* = 0.0057; FULL vs. Control *p* = 0.0001; scAAV-BDNF vs. GRAFT/NT-3 *p* = 0.048; FULL vs. GRAFT/NT-3 *p* = 0.001; FULL vs. GRAFT/ChABC *p* = 0.012). Rats receiving only scAAV-BDNF were statistically indiscernible from those receiving the full treatment (144.2 ± 35.6 vs. 157.2 ± 15.6) as both had abnormally high “grip” strength. Whereas the GRAFT/NT-3 treated group (76.7 ± 10.1 g) was not different from the CONTROL (60.1 ± 31.5 g), or the GRAFT/ChABC treated group (73.5 ± 10.2 g).

In summary, during normal activities, the majority of rats receiving scAAV-BDNF only or in combination with other treatments were unable to extend their wrist or open the digits of their paw. Further, the average force needed to overcome the wrist flexion was significantly increased.

This unexpected treatment-induced motor deficit, likely related to BDNF over-expression by cell grafts and spinal scAAV injection, did not allow the continuation of the originally planned experiment, and consequently, the focus shifted to the possible role of BDNF in the development of spasticity. Therefore, histological results are only reported for serotonergic fibers because of serotonin’s neuro-excitatory properties and potential role in the development of spasticity.

Because of the involvement of BDNF in nociception (21), the Hargreaves apparatus was used to analyze the possible treatment effect on the latency of injured forelimb withdrawal in response to a painful stimulus pre and post treatment. Only two groups, FULL and the GRAFT/NT-3, showed a significant reduction in latency of withdrawal of their preferred forelimb (*p* value: 0.016 and 0.038, respectively), indicating thermal hypersensitivity, in response to thermal stimulation when compared to baseline latency values (FULL: 9.0 vs. 11.4 s, GRAFT/NT-3 9.5 vs. 11.4 s). Other treatment groups did not show any significant change in withdrawal latency following lesion (data not shown). These results do indicate that within our experiment there was no obvious link between changes in nociception and the occurrence of spasticity-like symptoms. The lack of effects on thermo-sensitivity in some of the groups where BDNF was over-expressed could be based on various factors including effects of other treatment components or insufficient BDNF levels.

### Animal weights

**Figure 3:**
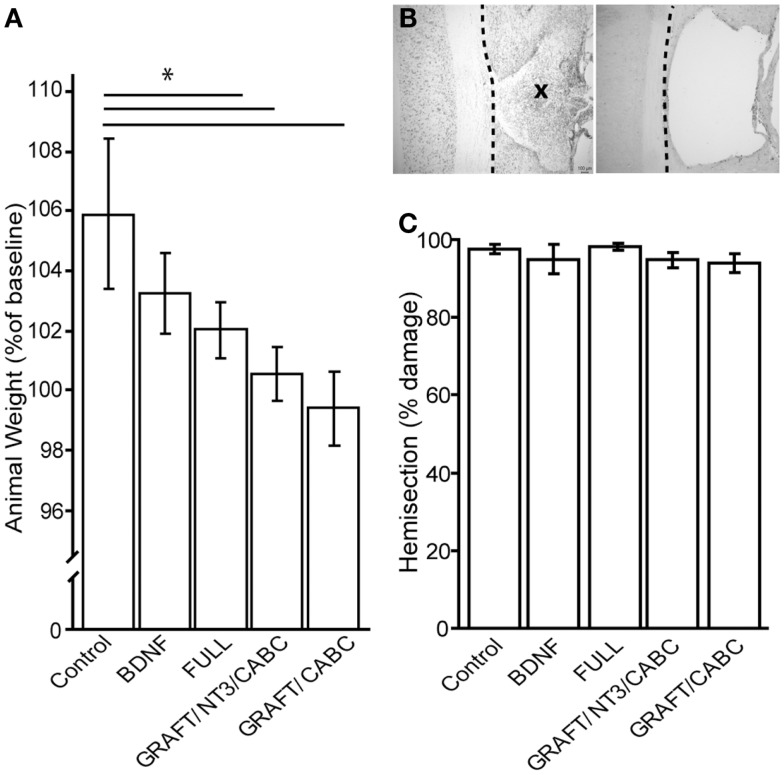
**Brain-derived neurotrophic factor application influences weight gain (A)**. Although baseline weight measurements were not statistically different between the treatment groups (not shown), after 9 weeks of recovery, the control group had gained significantly more weight than the FULL treatment group. Animals that received only scAAV-BDNF but no cell graft BDNF had moderate weight gain and were not significantly different from either group **(A)**. **(B)** Shows a representative example of a horizontal section through a lesion site with cell graft (left; indicated by an X) and without (right). Micrographs were taken at 100× magnification. There was no difference in lesion size among the groups **(C)**. *p < 0.05.

Because earlier findings indicate that BDNF reduces weight gain in rats (22, 23), decreased weight in the groups of animals receiving scAAVs expressing BDNF or a combination of grafts expressing BDNF and scAAVs expressing BDNF, might be one potential indicator for adverse effects of increased BDNF levels (Figure 3A).

When we compared the animal weights at the end of the experiment (i.e., 9 weeks post-injury) between the control rats that received a lesion and GFP expressing scAAV vectors (207 ± 1.7 g) and those that received only scAAV-BDNF (201 ± 1.1 g), we found a small, non-statistically significant decrease in weight gain. Rats that received the FULL treatment (which entails, among others, BDNF and NT-3 expressing cell grafts in addition to the injection of scAAV-BDNF) showed a significant difference (192 ± 2.3 g). GRAFT/NT-3 and GRAFT/ChABC treated group was not statistically different from any other treatment groups. However, it has to be kept in mind that this weight loss might have also been influenced by other treatment components such as rolipram injections in addition to the chronic BDNF over-expression by either the fibroblast cell graft or scAAV vectors.

### Lesion sizes

Lesion sizes were analyzed from reconstruction of serial sections (Figures 3B,C). Following our exclusion criteria, animals with lesions deviating from a complete hemisection by >10% were removed from this study (*n* = 4); a comparison of the lesion sizes between the groups showed no statistical difference between either group. The control group had only 2.2% (±1.1) tissue spared in comparison to 4.1% (±2.8) in the scAAV-BDNF only group, 1.1% (±1.4) in the FULL treatment group, 3.7 (±0.01) in the GRAFT/NT-3 group, or 4.12 (±0.01) in the GRAFT/ChABC group. Thus, variations in lesion size were only minor, and differences in functional outcome can therefore confidently be attributed to the different treatments.

### Sprouting of serotonergic fibers

**Figure 4:**
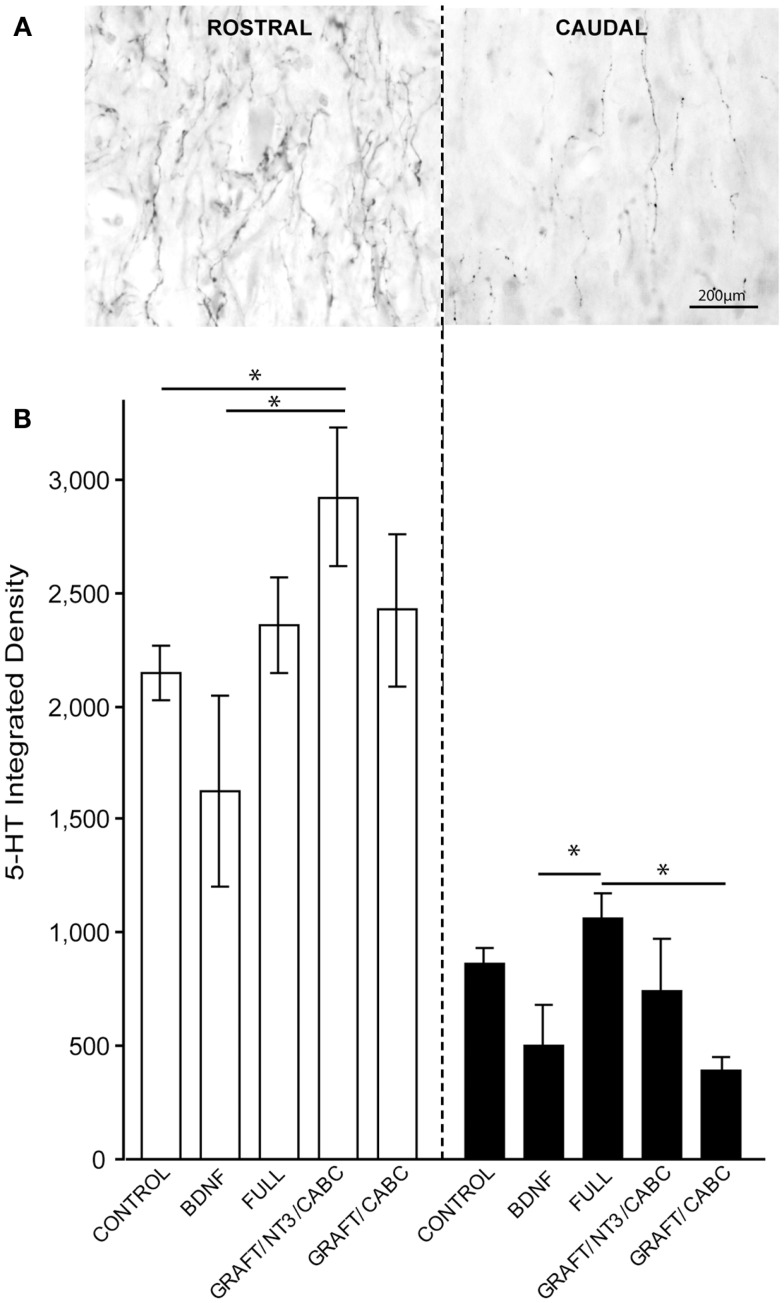
**5-HT fibers do not facilitate treatment-induced spasticity following SCI**. **(A)** Gray-scale images following 5-HT immunohistology show a high density of 5-HT fibers immediately rostral to lesion and their subsequent reduction following SCI caudal to the lesion. **(B)** Quantification of integrated 5-HT fiber density rostral and caudal to the lesion does not indicate significant influence of the BDNF treatment. Data presented as mean ± SEM, *p < 0.05.

Nine weeks after SCI, the density of 5-HT positive fibers both immediately rostral and caudal to the lesion site was quantified in order to visualize and compare the possible role of 5-HT fiber sprouting in the observed spasticity following SCI (Figure 4A).

The integrated density of 5-HT fibers was compared among all groups (Figure 4B). The highest values for 5-HT were observed in the GRAFT/NT-3 group (2923 ± 307) which is statistically significant when compared to the control group (2148 ± 117) and the scAAV-BDNF only group 1621 ± 418 (*p* = 0.0330, 0.0424, respectively). The average density value in the FULL treatment group was 2354 ± 212 and 2423 ± 337 in GRAFT/ChABC (not significant to controls).

Caudal to the lesion, 5-HT density was reduced and even though the FULL treatment group contained significantly more fibers than the scAAV-BDNF only group (*p* = 0.034; 1059 ± 116 vs. 503 ± 178; respectively), neither group was statistically different from the control group (863 ± 68). The FULL treatment group was however, significantly different from the GRAFT/ChABC group (1059 ± 116 vs. 395 ± 52; *p* = 0.0339), but not different from GRAFT/NT-3 group (1059 ± 116 vs. 739 ± 229). Thus, it appears unlikely that differences in the availability of serotonin contributed to the increase in spasticity of the treated groups.

### Antagonizing BDNF

In order to investigate the underlying mechanisms of the observed increase in spasticity-like symptoms of animals that received BDNF treatment either by scAAV vector expression or in combination with grafts expressing BDNF, we quantified the muscle activity elicited by overcoming the wrist flexion before and after the spinal injection of the BDNF antagonist TrkB-Fc.

Before injection of TrkB-Fc into the lesion site, wrist flexion was manually overcome three times in a row with an interval of about 3 s, which was repeated every 10 min following the TrkB-Fc injection for a total of 30 min. When this experiment was performed in rats without signs of spasticity (*n* = 3), there was no resistance to the stretch and no EMG activity was detected before or during the extension of the wrist into a horizontal position (i.e., 180°) even before TrkB-Fc was injected. Therefore, for this experiment only rats that demonstrated resistance and an EMG response in flexor muscles to stretching of the wrist (i.e., displayed spasticity-like symptoms) were included.

**Figure 5:**
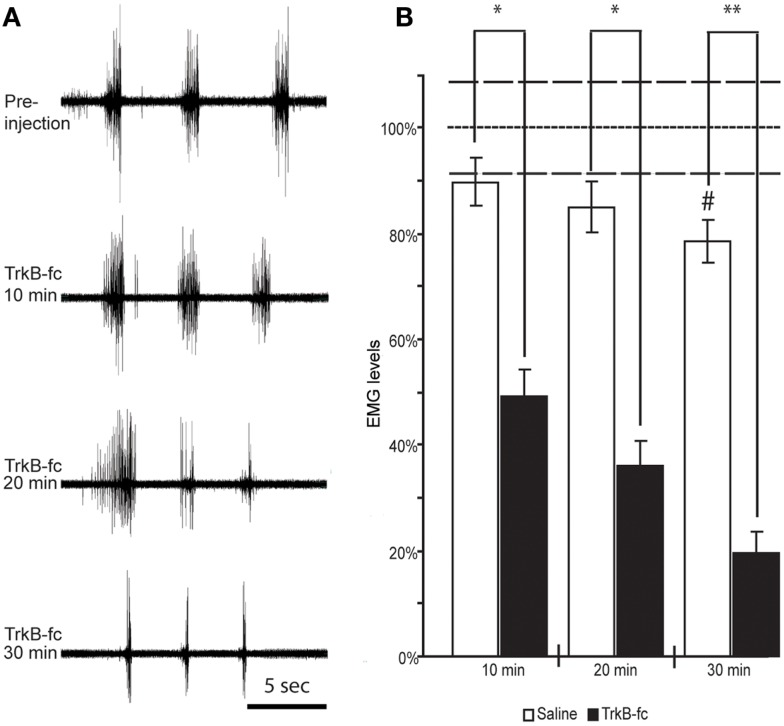
**Spinal BDNF antagonist reduces resistance to overcome wrist flexion in animals that exhibit spasticity-like symptoms**. EMG recordings from wrist flexor muscles of the forelimb ipsilateral to the lesion before and at three different time points following TrkB-Fc injection show a gradual decline in stretch-evoked EMG responses. Note the relative inactivity in the recording before and in between the stretch-evoked bursts **(A)**. When quantifying the EMG response and normalizing it to the values found in control animals under anesthesia only (did not receive Saline or TrkB-Fc injections; dotted line) we found a significant drop in EMG response at 10 min, and a continuous decline over the next 20 min when TrkB-Fc infusion was compared to saline injections **(B)**. ^#^Indicates a significant reduction of EMG response at 30 min post saline injection when compared to control animals that were under anesthesia only (dotted line; *p* = 0.0286). White columns represent rats that received saline, black columns represent rats that received the BDNF antagonist. Bars show means ± SEM; *p < 0.05. **p < 0.005. Dashed lines indicate the SEM for control anesthesia (dotted line).

When averaging the rectified EMG from all three stretches and comparing this value to values measured at 10 and 20 min following a spinal saline injection, we found in four animals with spasticity-like symptoms (one from the scAAV-BDNF, two from the FULL treatment group, and one from GRAFT/NT-3 group) only an insignificant decrease in EMG response over time (Figure 5). Only after 30 min, a significant decline of 11% was observed.

In contrast, in 10 animals with spasticity-like symptoms (two scAAV-BDNF and eight of the FULL treatment group) that received the BDNF antagonist injection, the EMG response dropped by 51 ± 9.8% starting as early as 10 min after TrkB-Fc injection and continued to decline to 64 ± 8.4% at 20 min and 80 ± 6.6% at 30 min. The difference in the stretch induced average and rectified EMG amplitude at each time point was statistically significant between saline and TrkB-Fc injected rats (Figure 5).

Interestingly, a significant effect of TrkB-Fc was also found in one out of two control treated rats that showed severe wrist flexion/spasticity, at 20 and 30 min post injection (*p* = 0.024 and 0.036, respectively).

## Discussion

Brain-derived neurotrophic factor is a prominent neurotrophic factor with a long history and a variety of effects. Such effects include the modulation of cell survival (ranging from protection to cell death), the enhancement of neurite outgrowth and axonal regeneration, the promotion of myelination, as well as the modulation of synaptic transmission, the post-injury immune response and neuronal excitability [reviewed in Ref. (8)]. Not surprisingly, BDNF also frequently makes it onto the list of potential SCI treatments [e.g., Ref. (11, 24–30)]. Considering the broad effects of BDNF, it is also not surprising that the mechanisms by which BDNF influences functional outcomes is sometimes difficult to discern. Understanding the mechanism by which a treatment influences functional recovery (or decline) is more challenging when several treatments are combined. A good example of this can be found in a recent report by Lu and colleagues (11), where a combined treatment not only resulted in enhanced plasticity of various descending fiber systems (e.g., serotonergic fibers) and axonal regeneration, but also in an undesired effect (i.e., increased occurrence of spasticity). Obviously it is of utmost importance to explore whether the increased spasticity was due to treatment-mediated augmentation of neurite growth and plasticity and/or possibly through an acute neuro-excitatory effect of BDNF (31, 32).

Another challenge of using BDNF for treating SCI is its well-established role in affecting sensory and nociceptive pathways in the spinal cord (21) and its ability to modulate glutamate receptors and sensitization (33, 34). Thus, a possible link between the observed spasticity-like symptoms found in the present study and neuropathic pain could be suggested. Yet, in this study no consistent change was found in thermo-sensitivity and it is unlikely that the signs of spasticity are related to increased pain. For example, it could be assumed that when assessing the grip strength, increased pain sensitivity would result in a withdrawal rather than an increase in grip strength.

Another potential mechanism involved in promoting spasticity could be aberrant serotonergic sprouting and the subsequent increased levels of serotonin, which could increase neuronal excitability (35, 36). In the current study, we did not find significant differences in the serotonergic innervation between the groups and demonstrate that a BDNF antagonist reduces spasticity within minutes. This indicates that an acute neuro-excitatory effect of permanent BDNF expression underlies the increase in signs of spasticity. The relatively fast effect of the TrkB antagonist also indicates that, although baseline activity in the flexed muscle was very low, a contracture [shortening of the muscle; Ref. (37)] was not involved in the observed spasticity-like symptoms.

It has to be kept in mind that spasticity-like symptoms were also observed in a small percentage of rats with spinal hemisection that received only GFP expressing viral vectors. It appears as though the continuous BDNF over-expression exacerbated this naturally occurring process by decreasing the threshold for the development of spasticity. Curiously, the application of the BDNF antagonist also reduced the spasticity in one control treated rat, indicating that BDNF signaling might also be involved in post-injury regulation of neuronal excitability. Indeed, increases in BDNF expression after SCI have been reported (38–40), which can contribute to increased neuronal excitability by reducing the amount of current required to reach threshold [i.e., rheobase; Ref. (41, 42)].

There are many possible mechanisms by which BDNF can exert its excitatory effects. For example, BDNF may increase calcium and sodium influx through TrkB signaling (43). Furthermore, activation of the TrkB-PLC pathway may raise the membrane potential through calcium-mediated channel opening (44). BDNF has also been reported to down-regulate the potassium-chloride co-transporter KCC2 (45), which down-regulates the inhibitory influence of gamma-aminobutyric acid (GABA) receptors. The most rapid and potent excitatory effects of BDNF, however, have been shown to involve Nav1.9, on a time scale similar to the action of glutamate (32).

Considering its neuro-excitatory properties, it is not surprising that over-expression of BDNF can also promote beneficial effects without promoting neuronal survival or regeneration. For example, Boyce et al. (41), showed that the injection of BDNF expressing vectors improved locomotor function in rats with complete spinal cord transections.

Effects of BDNF on synaptic connectivity have been described in dissociated cultures of early embryonic hippocampal neurons, where it increased the number of functional synaptic connections (46). At the same time, studies of long-term effects of BDNF in dissociated cortical cultures have suggested that BDNF decreases neuronal firing rate by reducing the strength of all excitatory inputs onto a given neuron (47). Bolton et al. (48) found that actions of BDNF underlying the increase in activity, involved enhancement of both excitatory and inhibitory synaptic transmission in parallel, but via distinct cellular mechanisms.

In the current study, it can only be speculated which neuronal population(s) responded to the BDNF antagonist, since the stretch induced spasticity-like reflex was decreased. From earlier studies (31, 49) it is likely that motoneurons play a major role in the development of spasticity. Generally speaking, following the administration of the BDNF antagonist we found a decline in EMG amplitude (see Figure 5 at 20 min) and a decline in burst duration in parallel to the decline in spasticity-like symptoms. This is in line with the idea that after SCI spasticity is related to the re-occurrence of plateau potentials in motoneurons allowing activity beyond the time of depolarization (49, 50).

In conclusion, although the permanent expression of BDNF using viral vectors may well promote certain aspects of recovery through neuro-excitatory mechanisms, the potential side effects would caution such application and point to the use of regulated expression systems to control BDNF expression (14). The general involvement of the TrkB receptor in the development of spasticity warrants further exploration.

## Conflict of Interest Statement

The authors declare that the research was conducted in the absence of any commercial or financial relationships that could be construed as a potential conflict of interest.
